# The Molecular Mechanism of *Clock* in Thermal Adaptation of Two Congeneric Oyster Species

**DOI:** 10.3390/ijms26031109

**Published:** 2025-01-27

**Authors:** Zhuxiang Jiang, Chaogang Wang, Mingyang Du, Rihao Cong, Ao Li, Wei Wang, Guofan Zhang, Li Li

**Affiliations:** 1 Key Laboratory of Breeding Biotechnology and Sustainable Aquaculture (CAS), Institute of Oceanology, Chinese Academy of Sciences, Qingdao 266071, China; jiangzx@qdio.ac.cn (Z.J.); ali@qdio.ac.cn (A.L.); gfzhang@qdio.ac.cn (G.Z.); 2 University of Chinese Academy of Sciences, Beijing 101408, China; dumy@qdio.ac.cn; 3 Shandong Province Key Laboratory of Experimental Marine Biology, Institute of Oceanology, Chinese Academy of Sciences, Qingdao 266071, China; wangcg@qdio.ac.cn (C.W.); rhcong@qdio.ac.cn (R.C.); wangwei@qdio.ac.cn (W.W.); 4 National and Local Joint Engineering Laboratory of Ecological Mariculture, Qingdao 266071, China; 5 Laboratory for Marine Biology and Biotechnology, Qingdao Marine Science and Technology Center, Qingdao 266071, China; 6 Oyster Industrial Technology Institute of Zhanjiang, Southern Marine Science and Engineering Guangdong Laboratory (Zhanjiang), Zhanjiang 524031, China; 7 Laboratory for Marine Fisheries Science and Food Production Processes, Qingdao Marine Science and Technology Center, Qingdao 266071, China

**Keywords:** *Clock*, temperature adaptation, oyster

## Abstract

Clock genes regulate physiological and metabolic processes by responding to changes in environmental light and temperature, and genetic variations in these genes may facilitate environmental adaptation, offering opportunities for resilience to climate change. However, the genetic and molecular mechanisms remain unclear in marine organisms. In this study, we investigated the role of a key clock gene, the circadian locomotor output cycles kaput (*Clock*), in thermal adaptation using DNA affinity purification sequencing (DAP-Seq) and RNA interference (RNAi)-based transcriptome analysis. In cold-adapted *Crassostrea gigas* and warm-adapted *Crassostrea angulata*, *Clock* was subject to environmental selection and exhibited contrasting expression patterns. The transcriptome analysis revealed 2054 differentially expressed genes (DEGs) following the knockdown of the *Clock* expression, while DAP-Seq identified 150,807 genes regulated by *Clock*, including 5273 genes located in promoter regions. The combined analyses identified 201 overlapping genes between the two datasets, of which 98 were annotated in public databases. These 98 genes displayed distinct expression patterns in *C. gigas* and *C. angulata* under heat stress, which were potentially regulated by *Clock*, indicating its role in a molecular regulatory network that responds to heat stress. Notably, a heat-shock protein 70 family gene (*Hsp12b*) and a tripartite motif-containing protein (*Trim3*) were significantly upregulated in *C. angulata* but showed no significant changes in *C. gigas*, further highlighting their critical roles in thermal adaptation. This study preliminarily constructs a thermal regulatory network involving *Clock*, providing insights into the molecular mechanisms of clock genes in thermal adaptation.

## 1. Introduction

The internal circadian clock regulates the physiological, metabolic, and behavioral processes of organisms, allowing them to cope with daily and seasonal environmental changes, such as light, temperature, and tidal cycles. Predicting and aligning circadian rhythms with these environmental fluctuations is crucial for maximizing the fitness of organisms [1,2]. Daily temperature fluctuations act as key synchronizing signals that help establish and maintain circadian rhythms [3]. Within a non-stressful range of environmental temperatures, the clock period remains relatively stable, a characteristic known as temperature compensation [4]. Under stressful temperature conditions (high and low temperature changes), the circadian clock actively responds to maintain biological activity [5]. Numerous studies have explored the underlying molecular mechanisms of the circadian clock under cold stress, revealing that it regulates downstream cold-responsive transcription factors, specifically C-repeat binding factors (CBFs), to exhibit circadian rhythms and enhance freezing tolerance [6,7]. In contrast, while some studies have observed the association between clock function and heat stress responses, the molecular signaling pathways and genetic basis underlying this relationship remain poorly understood.

The clock rhythm is governed by an internal molecular clock composed of transcriptional and translational feedback loops, involving a group of core clock genes: circadian locomotor output cycles kaput (*Clock*), basic helix–loop–helix ARNT-Like 1 (*BMAL1*), period (*Per*), and cryptochrome (*Cry*) [8,9]. The CLOCK-BMAL1 heterodimer binds to E-box DNA *cis*-elements to activate the transcription of PER and CRY. Complexes of PER and CRY proteins then translocate to the nucleus to inhibit this transactivation, establishing the feedback loop that drives the circadian rhythm [10]. As a key component of the circadian system, *Clock* regulates the downstream genes that control various physiological processes, including behavior, immunity, and metabolism [11]. Genetic variations in *Clock* have been shown to play a crucial role in the seasonal adaptation of cyprinid fish, possibly by altering transcription rates, which suggests that they may be helpful for organisms in adaptation to future climate change [12]. However, the function of *Clock* in thermal adaptation and the potential molecular basis have rarely been studied.

Many sessile marine mollusks, like oysters, mussels, and barnacles, inhabit the transitional zone where terrestrial and marine habitats meet [13,14,15]. The substantial and complex environmental cues, like water level, salinity, temperature, light, and food availability, affect the growth and behaviors and regulate the circadian rhythms of the species that inhabit these areas [16,17,18]. In addition to light and temperature cycles, these organisms must also adapt to tidal cycles, which result in the formation of combined tidal and daily rhythms [19]. Unlike the relatively regular temperature changes in terrestrial and marine environments, temperature fluctuations in these transitional (intertidal and supratidal) zones can be extreme, varying by as much as 10–20 °C over a single tidal cycle [19,20]. Studies have shown that most rhythmic gene expressions in intertidal organisms align with exposure to air during tidal cycles, potentially through circadian regulatory mechanisms that compensate for temperature changes [21]. As a widely distributed and economically important species in the intertidal zone, oysters also exhibit tidal and daily rhythms in their valve behavior [22,23]. In our previous resequencing and transcriptome analysis, we identified a strong selective sweep in the key rhythmic gene *Clock* in the cold-adapted *Crassostrea gigas* (*C. gigas*) and warm-adapted *Crassostrea angulata* (*C. angulata*), suggesting a functional link between *Clock* and long-term thermal adaptation [24]. The divergence in thermal tolerance between *C. gigas* and *C. angulata* has been well-documented [24], making these species ideal models for exploring the functional connection between rhythmic genes (*Clock*) and thermal adaptation at the molecular level.

In this study, we found that cold-adapted *C. gigas* and warm-adapted *C. angulata* exhibited opposite *Clock* expression patterns under heat stress, suggesting that the rhythmic gene *Clock* may contribute to thermal adaptation. Therefore, we integrated Daq-seq and RNAi-based transcriptional analyses to further explore the potential molecular mechanisms through which *Clock* may mediate thermal adaptation.

## 2. Results

### 2.1. Sequence and Expression Pattern Analysis

**Figure 1:**
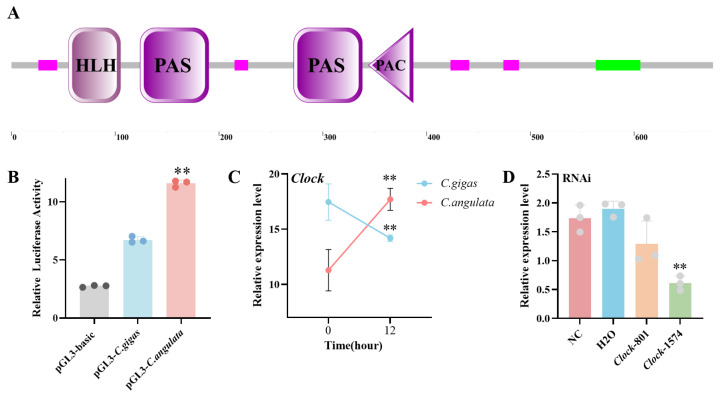
Functional and expression analyses of the *Clock* in *C. gigas* and *C. angulata*: (**A**) Structural domains of the CLOCK protein. The protein contains a basic helix–loop–helix (bHLH) domain, two PAS domains, and a PAC domain. The magenta blocks indicate conserved motifs, while the green block represents the transcriptional activation domain. The positions of each domain are denoted along the amino acid sequence. (**B**) The relative dual-luciferase reporter (DLR) values of the HEK293T cells transfected with the promoter of the *Clock* gene (n = 3). Control, *C. gigas*, and *C. angulata* in the legend represent the HEK293T cells that were introduced with the pGL3-basic plasmid ligated with empty pGL3 plasmid and about 2.0 k of the promoter sequence of *Hsp90* from *C. gigas* and *C. angulata*. The error bars represent SE. (**C**) The relative expression of *Clock* during short-term heat stress (37 °C, 12 h) in the gill tissues of *C. gigas* and *C. angulata* (n = 3). The blue line represents *C. gigas*, and the red line represents *C. angulata*. The error bars represent SE. (**D**) Two siRNAs’ (*Clock*-801 and *Clock*-1574) knockdown efficiency of *Clock*. The experiment groups were compared to the negative control (NC) and H_2_O treatments. Significant differences among groups were marked with ** *p* < 0.01.

SMART analysis revealed that *CgClock* contains a helix–loop–helix domain, two Per-Arnt-Sim (PAS) domains, and a PAS-associated C-terminal (PAC) domain, which is consistent with the structure observed in other species (Figure 1A). Our prior resequencing analysis revealed a strong selective sweep in the upstream noncoding region of the *CgClock* gene in *C. gigas* and *C. angulata*. To investigate this further, we evaluated the promoter activity of *CgClock* in both species. The results showed that the *CgClock* promoter activity was significantly higher in *C. angulata* than in *C. gigas* (*p* < 0.01) and demonstrated a greater positive transcriptional regulation in *C. angulata* (Figure 1B). Furthermore, the contrasting expression patterns, with *CgClock* being significantly upregulated (*p* < 0.01) in *C. angulata* but downregulated in *C. gigas*, suggest that *C. angulata* may increase transcriptional activity under heat stress, thereby upregulating *CgClock* expression and modulating the downstream genes to enhance thermal tolerance (Figure 1C). To identify potential downstream targets of *CgClock*, we performed RNAi experiments, and siRNA *Clock*-1574 significantly suppressed *CgClock* expression (*p* < 0.01), knocking down 65% as compared to the NC group (Figure 1D).

### 2.2. CgClock Regulate Genes and Functional Analysis

**Figure 2:**
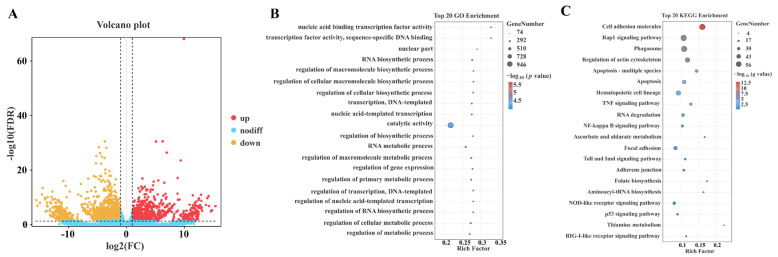
Analysis of differentially expressed DEGs following the knockdown of the *Clock* gene: (**A**) Volcano plot illustrating the distribution of DEGs based on the log2 fold change (FC) and −log10 false discovery rate (FDR). Red dots represent upregulated genes, yellow dots represent downregulated genes, and blue dots indicate non-differentially expressed genes. The thresholds for significance were set as |log2(FC)| > 1 and FDR < 0.05. (**B**) GO enrichment analysis of the top 20 biological processes associated with DEGs. The x-axis represents the enrichment factor, while the y-axis lists the enriched GO terms. The sizes of the dots correspond to the number of genes in each category, and the color gradient from blue to red represents the adjusted *p*-value, with red indicating higher significance. (**C**) KEGG pathway enrichment analysis of the top 20 pathways related to DEGs. The x-axis denotes the enrichment factor, and the y-axis displays the KEGG pathways. Dot size indicates the number of genes associated with each pathway, and the color gradient reflects the adjusted *q*-value, with red denoting higher statistical significance.

The RNAi-based transcriptome analysis generated approximately 428 million raw reads and 426 million clean reads, each with an average length of 150 bp (Table S1). Of these, 71.7% to 73.9% of the reads aligned to the oyster reference genome (Table S2). Principal component analysis revealed a clear separation between the RNAi group and the NC group (Figure S1). Differentially expressed genes (DEGs) were identified based on log2-fold change (log2FC), with log2FC ≥ 1 indicating upregulation and log2FC ≤ −1 indicating downregulation. As a result, a total of 859 upregulated and 1195 downregulated genes were identified in the RNAi group as compared to the NC group (Figure 2A). Gene ontology (GO) enrichment analysis showed that terms related to transcription factor activity, biosynthetic process, and metabolic process were most significantly enriched following the *Clock* knockdown (Figure 2B). KEGG enrichment analysis identified the most significantly enriched pathways, including cell adhesion molecules, Rap1 signaling pathway, phagosome, regulation of actin cytoskeleton, and apoptosis. Furthermore, some *Clock* gene-associated pathways such as the TNF, NF-kappa B, and p53 signaling pathways were also enriched (Figure 2C).

### 2.3. Bing Targets Analysis of CgClock in Oyster

**Figure 3:**
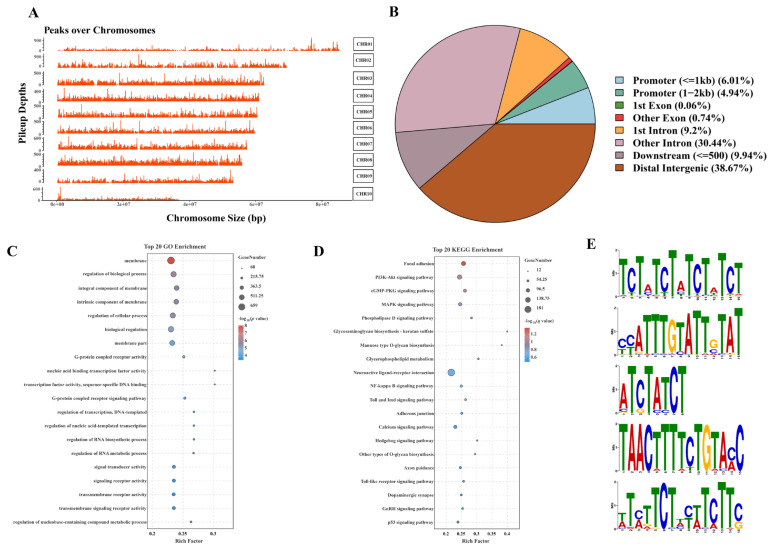
Genome-wide binding and functional annotation of *Clock* regulatory regions: (**A**) Distribution of DNA affinity purification sequencing (DAP-Seq) peaks across 10 oyster chromosomes. The y-axis represents the peak density, while the x-axis shows the genomic position (**B**). Distribution of DAP-seq peaks across genomic regions. The pie chart illustrates the percentage of DAP-seq peaks mapped to various genomic regions. The categories include promoter, 1st exon, other exon, 1st intron, other intron, downstream, and distal intergenic. (**C**) GO enrichment analysis of the top 20 biological processes associated with target genes. The x-axis represents the enrichment factor, while the y-axis lists the enriched GO terms. The sizes of the dots correspond to the number of genes in each category, and the color gradient from blue to red represents the adjusted *p*-value, with red indicating higher significance. (**D**) KEGG pathway enrichment analysis of the top 20 pathways related to target genes. The x-axis denotes the enrichment factor, and the y-axis displays the KEGG pathways. Dot size indicates the number of genes associated with each pathway, and the color gradient reflects the adjusted *q*-value, with red denoting higher statistical significance. (**E**) The top five enriched motifs in the *Clock* binding regions. The sequence logos depict the conserved DNA motifs identified in the DAP-Seq peaks.

Dap-Seq analysis was conducted to identify the downstream target genes of *CgClock* and their corresponding binding sites. Using MACS2, a total of 52,491 peaks with an average length of 360 bp were detected (*q* < 0.05) (Table S3). These peaks were unevenly distributed across all chromosomes, with the highest concentration observed on CHR01, CHR02, and CHR03 (Figure 3A). A total of 150,807 genes were identified according to the gene annotation, and genomic region analysis revealed that 49.14% of the peaks were located in distal intergenic regions, 39.64% in promoters, and 10.85% in other regions (Table S4) (Figure 3B). To identify the potential regulatory network of *CgClock*, we performed an enrichment analysis on 5273 identified genes in the promoter region. GO enrichment analysis showed significant enrichment in GO terms related to membranes and signal transduction, transcriptional activity, and gene expression regulation (Figure 3C). Among these, the membrane, the regulation of the biological process, and the integral component of the membrane were identified as the most enriched terms. KEGG pathway analysis revealed that the identified pathways belong to three major categories: signal transduction, cellular structure, and immunity. Notably, all the signal transduction pathways such as the PI3K-Akt signaling pathway, the MAPK signaling pathway, and the NF-kappa B signaling pathway are associated with thermal adaptation in oysters (Figure 3D). Additionally, MEME-ChIP was utilized to identify enriched DNA sequence motifs near the apex of the DAP-seq peaks in the *C. gigas* genome. This analysis revealed 12 significant motifs, including six MEME motifs, three DREME motifs, and three CentriMo motifs. The sequences of the top five significant motifs are presented in Figure 3E.

### 2.4. Functional Analysis of CgClock in Thermal Adaptation

**Figure 4:**
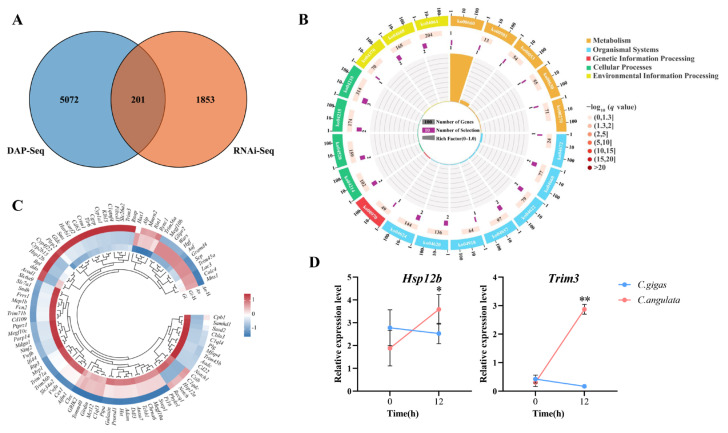
*Clock*-regulated thermal adaptation networks in *C gigas* and *C. angulata*: (**A**) Venn diagram showing the overlap between the genes identified by DAP-Seq and RNA-Seq. (**B**) Functional classification of the 201 overlapping genes based on KEGG pathway enrichment analysis. The circular plot categorizes the pathways into five functional groups: metabolism, organismal systems, genetic information processing, cellular processes, and environmental information processing. The sizes of the bars indicate the number of genes enriched in each pathway, and the color gradient represents statistical significance (−log10(*q*-value)). (**C**) Heatmap and hierarchical clustering of the 98 annotated overlapping genes with differential expression patterns. The heatmap highlights the opposing gene expression trends in *C. gigas* and *C. angulata* under heat stress. Blue and red gradients represent low and high expression levels, respectively. (**D**) The relative expression of *Hsp12b* and *Trim3* during short-term heat stress (37 °C, 12 h) in the gill tissues of *C. gigas* and *C. angulata* (n = 3). The blue line represents *C. gigas*, and the red line represents *C. angulata*. The error bars represent SE. Significant differences among the groups were marked with * *p* < 0.05, ** *p* < 0.01.

To investigate the direct regulation of the downstream target genes of *CgClock*, we performed a combined analysis of RNAi-based transcriptome-seq and *CgClock* DAP-seq, and a total of 201 genes were identified in the significant changes in both groups (Figure 4A). According to gene annotation, 98 genes were matched to the public database, and KEGG enrichment analysis showed that nine signal transduction-related pathways, four metabolism-related pathways, and four apoptosis-related pathways were significantly enriched (Figure 4B), which indicates that these pathways may mediate the divergence thermal adaptation of *C. gigas* and *C. angulata*. To further explore the regulatory network, we reanalyzed our previous transcriptome data [25] and examined the expression patterns of these 98 key genes under heat stress in both oyster species. The results showed that most of the *CgClock* direct regulatory genes in *C. gigas* and *C. angulata* showed different expression patterns (Figure 4B), which is consistent with the opposite expression of *CgClock*. Notably, a heat-shock protein 70 family gene (*Hsp12b*) and a tripartite motif-containing protein (*Trim3*) were significantly upregulated in *C. angulata* but showed no significant changes in *C. gigas*, which may be regulated by the divergency expression pattern of *CgClock* in the two oysters.

## 3. Discussion

As a key gene in circadian rhythm regulation, *Clock* regulates various physiological processes including cell proliferation, DNA damage repair, and metabolism to maintain daily life activities in organisms [9,11]. In oysters, *Clock* contains a conserved PAS-HLH transcription-factor domain, which is vital for dimer formation with BMAL1, and this indicates the similarity of its biological function with that of other organisms. In our study, the opposite expression patterns of *Clock*, with downregulation in cold-adapted *C. gigas* and upregulation in warm-adapted *C. angulata*, indicate its significant role in temperature adaptation. This may be associated with the higher transcriptional activity in the promoter region of *Clock* in *C. angualta*, which has been identified as a key factor in gene expression regulation [26]. Moreover, circadian clock proteins have been identified with temperature compensation functions to enable robust and precise timing under changing environmental temperatures [27,28], and the heat-responsive *Clock* in *C. angulata* suggests its involvement in temperature compensation under heat stress. In the circadian regulation process, the increased *Clock* expression can promote the formation of the BMAL1:CLOCK complex, which can further activate the transcription of the downstream genes and regulate the physiological processes [5,29,30]. Therefore, the upregulation of *Clock* in *C. angulata* may activate the downstream pathways to resist heat stress.

After the knockdown of *Clock* in oysters, more genes were downregulated, which is consistent with its role as a positive regulatory factor in the cell cycle [11]. Through the GO Enrichment Analysis for DEGs, the transcription factor activity, biosynthetic process, and metabolic process were found to be significantly enriched. As the core member in the circadian feedback loop, the upregulation of *Clock* accelerates the formation with another transcriptional factor, *Bmal1*, and then positively regulates and activates the expression of other rhythm genes, including period (*Per1*, *Per2* and *Per3*) and cryptochrome (*Cry1* and *Cry2*) [31,32]. Furthermore, many of the activated rhythm genes were identified as transcription factors themselves, which can mediate metabolic and physiological processes by driving the transcription and translation of numerous downstream genes [33]. Therefore, *Clock* in oysters may regulate a wide range of physiological processes through the activation of downstream transcription factors. Additionally, the significant enrichment of pathways such as TNF, NF-kappa B, and p53 signaling pathways further confirms the role of *Clock* in regulating fundamental physiological processes. Both the TNF and p53 pathways have been implicated in regulating cell apoptosis and survival [34,35], with the p53 pathway also involved in DNA repair, preventing and correcting DNA damage [36]. This suggests a crucial role for *Clock* in oysters under stress conditions. Notably, TNF-α can inhibit CLOCK-BMAL1-induced activation of the E-box regulatory elements, thus regulating the expression of clock genes [37]. The p53 pathway is also activated by the circadian gene *Bmal1*, further regulating apoptosis [38], suggesting the importance of *Clock* in regulating cell apoptosis. Interestingly, the NF-κB pathway was significantly enriched among the *Clock* downstream target genes identified by DAP-Seq, which demonstrates a direct regulatory relationship between the NF-κB pathway and *Clock* in oysters. Our previous study found that the MAPK-NF-κB cascade exhibits divergent thermal responses and adaptation patterns between *C. gigas* and *C. angulata* [39]. Specifically, the MAPK pathway showed stronger activation in response to higher environmental temperatures in *C. angulata*, further activating cell survival, fatty acid metabolism, protein translation, and antioxidant gene expression to resist heat stress [39]. Therefore, the opposite expression pattern of *Clock* in these two species may influence heat tolerance through the regulation of the MAPK pathway, a phenomenon referred to as “temperature compensation” in circadian genes [29,40,41].

In the combined analysis of the transcription and DAP-seq data, 201 genes were identified, of which 98 were annotated. Using the transcriptomic data from *C. gigas* and *C. angulata* under heat stress that were obtained from the previous study, we found that most of these 98 genes exhibited divergent expression patterns, suggesting a direct regulatory relationship with *Clock*. This highlights the critical role of these genes in mediating the thermal divergence between the two oyster species. Among these genes, the *Hsp70* family gene *Hsp12b* and *Trim3* exhibited opposite repression patterns, with *Hsp12b* and *Trim3* upregulated in *C. angulata*. This suggests that *Clock* may mitigate the effects of heat stress by regulating the heat shock proteins and the ubiquitination process. Under environmental stress, heat-damaged or misfolded proteins accumulate within the cells [42], and molecular chaperones, particularly heat shock proteins, assist in refolding these proteins into their native states [43,44,45]. However, if the damage exceeds the repair capacity, these proteins are degraded by proteases through the Ub–proteasome pathway [46]. Among the heat shock proteins, *Hsp70* has been widely studied in thermal adaptation research; its substrate-binding domain (SBD) binds to damaged proteins, protecting them from denaturation or aggregation [47]. Furthermore, the high plasticity expression of *Hsp70* is associated with short-term and long-term thermal adaptability in organisms [48,49], which is consistent with the upregulation in *C. angulata*. In oysters, the remarkable genomic expansion and high plasticity expression of *Hsp70* under heat stress suggest the key to *Hsp70*’s adaptation to environments with fluctuating temperatures [23]. Moreover, several heat shock proteins (*Hsp20*, *Hsp40*, *Hsp60*, *Hsp70*, *Hsp90*) in *C. gigas* and *C. angulata* exhibit divergent expression patterns [50], and *Hsp70* is under subject to environmental (temperature) selection during local adaptation in warm and cold habitats [24]. Therefore, the upregulation of *Hsp12b* in *C. angulata* may help protect proteins from heat-induced damage, thereby enhancing thermotolerance of *C. angulata*. Another gene TRIM3 was identified as a ubiquitin ligase that targets ACTG1 for proteasomal degradation [51]. Although it is rarely reported as having related thermal adaptation, it can interact with P53 to promotes the formation and degradation of ubiquitin chains and thus promote apoptosis [52]. The upregulation of *Trim3* may contribute to the degradation of excessively damaged cells in *C. angulata* and, together with the protective effect of *Hsp12b*, enhance its heat tolerance.

In summary, our study reveals that the *Clock* gene plays a crucial role in thermal adaptation in *C. gigas* and *C. angulata*, with opposite expression patterns observed under heat stress. *Clock* regulates various physiological processes including heat shock response and ubiquitination through the activation of downstream genes such as *Hsp12b* and *Trim3*. These genes are involved in protein repair and degradation, respectively, and contribute to heat tolerance. The divergent expression patterns of *Clock* and its targets highlight their roles in mediating thermal divergence between the two oyster species, underscoring the importance of circadian regulation in environmental adaptation.

## 4. Materials and Methods

### 4.1. Experimental Animals

The wild *C. gigas* and *C. angulata* were collected from their natural habitats: Qingdao (35°44′ N, 5–26 °C) and Xiamen (24°33′ N, 15–28 °C), respectively. To minimize the influence of local environmental conditions, we conducted a one-generation common garden experiment prior to the heat shock experiment. Briefly, juvenile F1 progeny (8 months old) of each species were separated into two groups: one group was deployed at Qingdao and the other group was deployed at Xiamen. Two months later, 10-month-old oysters were sampled from each site and collected to the laboratory for subsequent studies. Then, *C. gigas* and *C. angulata* were acclimated at 22–26 °C and 31 ±  1‰ (optimum salinity range 25–35‰) seawater for one week before the experiment.

For the heat shock experiment, the experimental group was exposed to a sublethal temperature of 37 °C for 12 h, while the control group remained in seawater at 18 ± 2 °C. Each group of Gi (*C. gigas*), Gi-H (*C. gigas* under heat stress), An (*C. angulata*), and An-H (*C. angulata* under heat stress) included 15 oysters that were sampled. The gill tissues were flash-frozen in liquid nitrogen and stored at −80 °C for subsequent analysis.

### 4.2. Sequence Analysis of the CgClock

Based on the oyster genome assembly (GCA_011032805.1), we amplified the Open Reading Frame (ORF) sequence of *CgClock* and analyzed its structural domains using the SMART tool (https://smart.embl.de, accessed on 16 October 2024).

### 4.3. RNAi Experiment

The small interfering RNA (siRNA) was synthesized by GenePharma (Shanghai, China), and the sequences are provided in Table S5 The siRNA injection procedure followed our previous study [39]. Briefly, oysters were acclimated for one week before being anesthetized. Each oyster was then injected with the corresponding reagent: 100 μL of siRNA (0.33 OD) for the siRNA group, 100 μL of a noncoding strand (0.33 OD) for the NC group, and 100 μL of seawater for the water group. Based on the results of the pilot experiment, *Clock*-1574 was identified as the effective siRNA and selected for the formal experiment. For the RNA extraction used in the RNA-seq, 15 individuals from both the NC and siRNA groups were injected with 100 μL of the noncoding strand or *Clock*-1574, respectively, and gill tissues were collected 24 h post-injection.

### 4.4. qRT-PCR Experiment

Gill tissues were collected from 15 individuals from both *C. gigas* and *C. angulata*, and TRIzol reagent (Vazyme Biotech, Nanjing, Chnia) was applied to extract the total RNA from the samples. First-strand cDNA synthesis was performed with HiScript III RT SuperMix for qPCR (Vazyme Biotech). Primers (Table S6) were designed with Primer 5.0 software and synthesized by RuiBiotech. Each experimental group consisted of three biological replicates, formed by pooling cDNA from five oysters in equal proportions, followed by three technical replicates. Ef-1α was used as the internal reference gene. qPCR was conducted using Taq Pro Universal SYBR qPCR Master Mix (Vazyme Biotech), and the relative gene expression was analyzed using the 2^−ΔΔCT^ method [53].

### 4.5. Dual-Luciferase Reporter Assay

The *Clock* promoter regions from *C. gigas* and *C. angulata* were amplified via PCR and inserted into the pGL3-basic vector (MiaoLing Plasmid Platform, Wuhan, China) using the ClonExpress II One Step Cloning Kit (Vazyme Biotech). HindIII restriction sites were chosen for vector construction, employing restriction enzymes from New England Biolabs. HEK293 cells were cultured according to the standard protocols [54]. For transfection, 480 ng of the *Clock* promoter-containing plasmid and 20 ng of the pRL-TK Renilla luciferase plasmid (per well) were co-transfected using Lipofectamine 3000 (Invitrogen, Carlsbad, CA, USA). Luciferase activities were measured using the dual-luciferase reporter assay system (Promega, Madison, WI, USA) and quantified with a Varioskan Flash multimode reader (ThermoFisher Scientific, Waltham, MA, USA). To ensure accurate comparisons, the Firefly luciferase activity was normalized to Renilla luciferase activity.

### 4.6. RNA-Seq Analysis

RNA was isolated from the gill tissue of *C. gigas* and *C. angulata*, and for each group, five individuals were pooled as one sample for RNA-seq. Library preparation, sequencing, and raw data analysis were conducted as described in our previous study [39]. Genes with a *q*-value ≤ 0.05 and a log2 fold change ≥ 1 were considered significantly differentially expressed. The differentially expressed genes (DEGs) were subjected to enrichment analysis using GO (http://geneontology.org, accessed on 10 August 2024) and KEGG (http://kobas.cbi.pku.edu.cn/genelist, accessed on 10 August 2024) tools, with a significance threshold of *p* ≤ 0.05.

### 4.7. DNA Affinity Purification Sequencing (DAP-Seq)

The full-length coding sequence (CDS) of *Clock* was amplified using the ClonExpress II One Step Cloning Kit (Vazyme Biotech) and then integrated into the pFN19K HaloTag^®^ T7 SP6 Flexi^®^ Vector. The constructed *Clock*-HaloTag vector was sent to Gene Denovo Biotechnology (Guangzhou, China) for the in vitro co-expression and sequential purification of the proteins. Genomic DNA were extracted from the gill tissues of *C. gigas* and fragmented by sonication to generate fragments approximately 200 bp in length. The fragments were amplified through PCR to obtain the qualitied library and then incubated with *Clock* proteins immobilized on magnetic beads. After the PCR amplification and bead purification, we utilized the Illumina HiSeqTM4000 to sequence the obtained fragments, removed the adapters and low-quality reads of the raw data to obtain clean reads, and then aligned to the *C. gigas* genome. For bioinformatics analysis, deepTools (version: 3.2.0) was used to quantify the regions from the transcriptional start site (TSS) to the transcriptional termination site (TES), as well as 2 kb intervals upstream and downstream. MACS2 was employed to identify enriched regions, with p-values calculated using Poisson distribution. If *q* < 0.05 regions were defined as peaks, the peak-related genes were annotated using the ChIPseeker R package, and their distribution across genomic regions was then analyzed. Motif prediction and analysis were conducted using MEME and DREME.

## Data Availability

The cDNA sequence of *CgClock* was deposited in the GenBank with accession number PQ675616. The raw sequencing data of transcriptome in the RNAi experiment and DAP-Seq in this study have been deposited the Sequence Read Archive (SRA) BioProject under the accession numbers PRJNA1193684 and PRJNA1193685, respectively.

## Supplementary Materials

The following supporting information can be downloaded at https://www.mdpi.com/article/10.3390/ijms26031109/s1.
