# Supplementary material for: The Molecular Mechanism of Clock in Thermal Adaptation of Two Congeneric Oyster Species
Source: Int J Mol Sci. 2025 Jan 27;26(3):1109. doi: 10.3390/ijms26031109 (PMC11817431; doi:10.3390/ijms26031109)
Supplement: Supplementary file 1 [file ijms-26-01109-s001.zip › Supplemantary material.pdf]

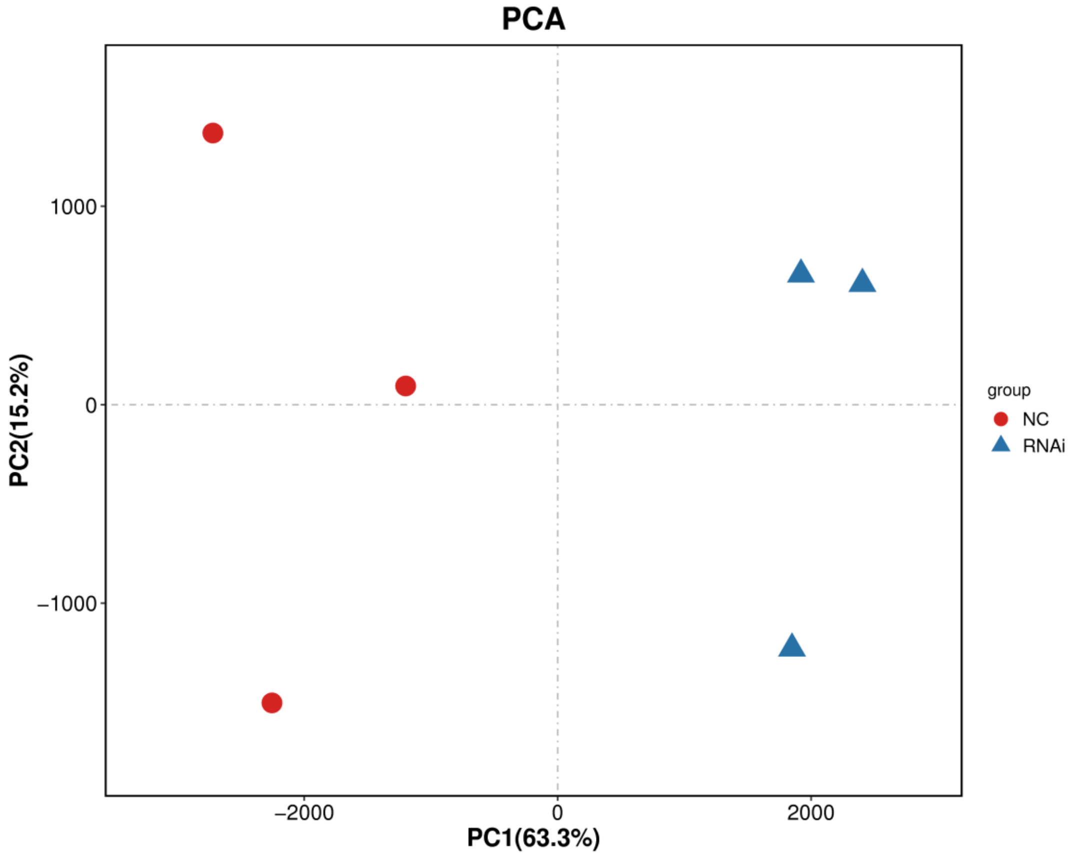

Figure S1. Principle component analysis of genes expression between the two groups. The points represent scores of biological replicates (n = 3).

Table S5. SiRNA of the RNA interference assay.

Name	Sense (5'-3')	Antisense (5'-3')
Clock-801	CCCAGUUUAUCCGGGAAAUTT	AUUUCCCGGAUAAACUGGGTT
Clock-1574	GGAGCUGACAAUGUAUCAUTT	AUGAUACAUUGUCAGCUCCTT

Table S6. Primer sequences for qRT-PCR.

Abbreviation	Sequence of primers (5'-3')
Clock-F	CTCCCAAGCCGAAGTGAAAG
Clock-R	ATGGTTGACTTTAGGACGGT
Hsp12b-F	AAGCCCATCATCTGGTTCTG
Hsp12b-R	CTTTGTAGTTTCTGTGGGTT
Trim3-F	CCCTCACATCCTTTCTCCCT
Trim3-R	CGTGCCAGTGTATGGTCAGT
Ef-1α-F (*C. gigas*)	AGTCACCAAGGCTGCACAGAAAG
Ef-1α-R (*C. gigas*)	TCCGACGTATTTCTTTGCGATGT
Ef-1α-F (*C. angulata*)	TTCCCAGCAAGCCTATGT
Ef-1α-R (*C. angulata*)	GCTCAGCCTTCTCAACCTC
